# Shifts of the point-of-change can be attributed to a lower mechanical cost of motor execution

**DOI:** 10.1007/s00221-020-05781-3

**Published:** 2020-03-26

**Authors:** Christoph Schütz, Thomas Schack

**Affiliations:** 1 Faculty of Psychology and Sports Science, Bielefeld University, PO Box 10 01 31, 33501 Bielefeld, Germany; 2 Cluster of Excellence Cognitive Interaction Technology, Bielefeld University, Bielefeld, Germany; 3 CoR-Lab, Research Institute for Cognition and Robotics, Bielefeld University, Bielefeld, Germany

**Keywords:** Motor planning, Motor hysteresis, Reaching, Handedness, Cognitive cost, Mechanical cost

## Abstract

In a previous study on hand selection in a sequential reaching task, the authors showed a shift of the point-of-change (POC) to the left of the midline. This implies that participants conducted a number of contralateral reaches with their dominant, right hand. Contralateral movements have longer planning and execution times and a lower precision. In the current study, we asked whether lower mechanical costs of motor execution or lower cognitive costs of motor planning compensated for these disadvantages. Theories on hemispheric differences postulate lower mechanical costs in the dominant hemisphere and lower cognitive costs in the left hemisphere (independent of handedness). In right-handed participants, both factors act agonistically to reduce the total cost of right-handed reaches. To distinguish between the cost factors, we had left- and right-hand-dominant participants execute a sequential, unimanual reaching task. Results showed a left-shift of the POC in the right-handed and a right-shift in the left-handed group. Both shifts were similar in magnitude. These findings indicate that only the mechanical cost of motor execution compensates for the disadvantages of the contralateral reaches, while the cognitive cost of motor planning is irrelevant for the POC shift.

## Introduction

When opening our sock drawer in the morning, we are blissfully unaware of the series of sensorimotor transformations our central nervous system has to perform to translate the retinal image of the drawer handle into a muscle activation pattern that guides our hand to the handle’s location. Due to these transformations, the creation of a reaching movement plan is associated with a cognitive cost. This cognitive cost becomes visible when participants carry out repetitive tasks. When opening a series of drawers with cylindrical handles, participants persist in their previous posture, i.e., a more pronated posture in a descending and a more supinated posture in an ascending sequence (Schütz and Schack 2013; Schütz et al. 2011).

The posture adopted at each drawer, therefore, depends on participants’ movement history. This *motor hysteresis effect* (Kelso et al. 1994) indicates that we do not create a new motor plan from scratch for each drawer, but instead partially reuse the previous motor plan (Schütz et al. 2016). According to the *plan-modification hypothesis* (Rosenbaum and Jorgensen 1992), plan reuse reduces the cognitive cost of motor planning. Motor hysteresis effects have also been demonstrated in binary posture selection tasks (e.g., over- vs. underhand grasp; Rosenbaum and Jorgensen 1992; Weigelt et al. 2009). In these binary tasks, reuse of the previous posture/motor plan is restricted to a *range of indifference*, in which participants are equally content with both alternatives (Rosenbaum and Jorgensen 1992).

Motor hysteresis has not only been found for posture selection, but for limb selection as well (Rostoft et al. 2002; Weiss and Wark 2009). Rostoft et al. (2002) had children catch a ball that rolled towards them on an inclined tabletop, choosing the hand that felt most natural. Ball presentation was varied in a left- or rightward progression. Results revealed a hysteresis effect for hand selection: the point-of-change (POC) between hands was shifted more to the left in a rightward and more to the right in a leftward progression. The mean POC was shifted to the left of the mid-line. This indicates that the (predominantly right-handed) children performed contralateral reaches with their dominant, right hand.

Contralateral reaches require a transfer of information across the corpus callosum. They have several disadvantages, such as longer reaction times, movement duration, and a higher positional error (Carey et al. 1996; Carson et al. 1992; Hoptman and Davidson 1994). The left-shift of the point-of-change suggests that some reduction in movement cost compensates for these disadvantages. According to the *cost-optimization hypothesis* (Schütz et al. 2016), total movement cost is the sum of the cognitive cost of motor planning and the mechanical cost of motor execution. Thus, a lower cognitive or lower mechanical cost (e.g., resulting from hemispheric lateralization; cf. MacNeilage et al. 2009) could be responsible for the left-shift of the POC.

Janssen et al. (2009, 2011) tested left- and right-handed participants in an anticipatory reaching task. Anticipatory planning was more pronounced for the right hand, independent of handedness. From this result, the authors concluded that motor planning is a specialized function of the left hemisphere. Such a specialization should reduce the cognitive cost of planning for the right hand. Neuroimaging studies found that long-term practice resulted in a reduced or more focused activation of M1 and the premotor areas during motor execution, which has been attributed to an increased efficiency of the underlying neural circuits (Haslinger et al. 2004; Hund-Georgiadis and von Cramon 1999; Jäncke et al. 2000; Krings et al. 2000; Meister et al. 2005).

Picard et al. (2013) trained monkeys for up to 6 years in a sequential reaching task and then measured the metabolic ([^14^C]2-deoxyglucose uptake) and neural (single-neuron recording) activity in M1. Results showed a significant reduction in metabolic activity after long-term training, but no change in neural activation, indicating an increase in efficiency of the neural circuits: to generate the motor activation necessary for the execution of the task, less synaptic and less metabolic activity was required. Therefore, if the left hemisphere was specialized in motor planning as stated by Janssen et al. (2009, 2011), the cognitive cost of planning should be lower in the right hand, independent of handedness.

The mechanical cost of motor execution has also been found subject to hemispheric lateralization. According to the *dynamic-dominance hypothesis* (Sainburg 2002), the dominant hemisphere has superior control of inertial dynamics, as muscle torques and interaction torques act agonistically to create the movement (Coelho et al. 2013). This reduces the total required torque impulse (Bagesteiro and Sainburg 2002) and, thus, the mechanical cost of the movement (Sainburg 2014). The non-dominant hemisphere, on the other hand, has a better impedance control created by co-activation of antagonistic muscles. This co-activation in the non-dominant limb provides better stability against perturbations, but increases the mechanical cost of the movement (Sainburg 2014).

In right-hand-dominant participants, both cost factors act agonistically to reduce the total cost of the movement for right-handed reaches, as one would expect a lower cognitive cost in the right hand (Janssen et al. 2009, 2011) and a lower mechanical cost in the dominant hand (Sainburg 2002), which is also the right. The lower total cost of right-handed reaches should shift the POC to the left of the mid-line. Indeed, the children in the study by Rostoft et al. (2002) were predominantly right-handed.

To test whether one or both cost factors were responsible for the left-shift of the POC, in the current study, we replicated the catching task by Rostoft et al. (2002) with a right-handed and a left-handed, adult participant group. Participants executed randomized (unaffected by motor hysteresis, cf. Schütz et al. 2011) and ordered sequences of unimanual catching trials. Based on the findings of Rostoft et al., we expected a significant hysteresis effect in both participant groups, i.e., a shift of the POC more to the left in a rightward and more to the right in a leftward progression. More importantly, however, we were interested in the mean shift of the POC in the left-handed group: in left-handed participants, the cognitive and mechanical costs act antagonistically, with a lower cognitive cost in the right hand but a lower mechanical cost in the dominant, left hand.

If the cognitive cost alone was relevant to the shift of the POC, we would expect a left-shift in both the right- and the left-handed group. If only the mechanical cost was relevant, we would expect a left-shift in the right-handed, but a right-shift in the left-handed group. If both cost factors were relevant, we would expect a smaller shift in the left-handed than in the right-handed group, as cost factors act agonistically in right-hand-dominant participants (sum of both effects) but antagonistically in left-hand-dominant participants (difference of both effects). For example, if cognitive cost was slightly less relevant than mechanical cost, we would expect a right-shift of the POC in the left-handed group, but of a considerably smaller magnitude than the left-shift in the right-handed group.

In general, the consistency of hand selection has been found to be lower for left-handed participants. In hand preference questionnaires, left-handers show a lower tendency towards the extreme responses (Papadatou-Pastou et al. 2013). More importantly, in real motor tasks (e.g., puzzling, block stacking), left-handers use their non-dominant hand significantly more often than right-handers (Gonzalez et al. 2007; Stone et al. 2013). Gonzalez and Goodale (2009) tested left-/right-handers in a block stacking task and found that the left-handers were split into two subgroups: left-left-handers, who used their left hand significantly more often than right-handers, and left-right-handers, whose hand selection did not differ from the right-handers. This indicates that the variance of hand preference in left-handers is higher than in right-handers, which has to be taken into account when analyzing the results.

We also measured reaction times (RTs) under three different planning demands: (1) if no re-planning was required, (2) if some re-planning was required, and (3) if full planning was required. Higher planning costs are commonly associated with longer RTs (Diedrichsen et al. 2001, 2003; Spijkers et al. 1997). Thus, we expected increasing RTs from conditions (1) to (3), resulting in a significant linear contrast. RTs were measured separately for the left and right hands. A number of studies observed shorter RTs for left-handed reaching (Boulinguez et al. 2000; Carson et al. 1995, 1992), independent of the handedness (Boulinguez et al. 2001). We, therefore, expected longer RTs in the right hand.

## Materials and methods

### Participants

Fifty-four students (30 females, 24 males, age 23.6 ± 3.2 years) from Bielefeld University participated in the experiment in exchange for course credit or 5€. Participants were recruited in two groups based on their self-reported handedness. Twenty-seven participants saw themselves as right-handed (self-report). All of these 27 were right-handed [handedness score (HS) 0.98 ± 0.06] according to the revised Edinburgh Inventory (Oldfield 1971). The remaining 27 participants saw themselves as left-handed (self-report). Of these, 19 were left-handed (HS − 0.76 ± 0.15) and eight ambidextrous (HS − 0.23 ± 0.14) according to the revised Edinburgh Inventory. Each participant read a detailed set of instructions on the task and provided written informed consent before the experiment. The study was approved by the local ethics committee and in accordance with the latest revision (World Medical Association 2013) of the 1964 Declaration of Helsinki.

### Apparatus

**Fig. 1:**
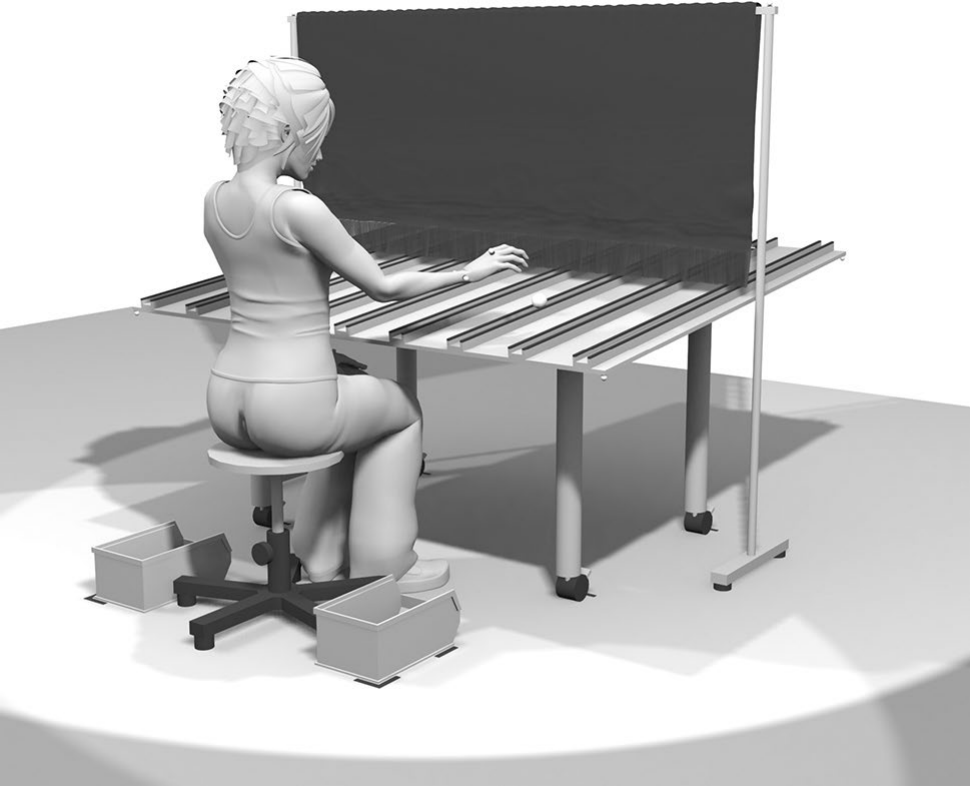
Schematic of the experimental setup. Golf balls roll towards the participants in one of nine lanes mounted on a tilted tabletop. Lane spacing is scaled to the arm span of the participants. Participants catch each ball with the hand that feels most natural. A black curtain obscures the location of the ball during setup

The apparatus used was a table with a tilted surface (1450 × 1650 mm, see Fig. 1) and a slope of 140 mm [difference between the low, front edge (700 mm) and the high, back edge (840 mm)]. On top of the table, nine wooden lanes (1450 × 50 × 30 mm each) were mounted. Lanes were covered with foam rubber to suppress the rolling noise of the ball. The positions of the lanes could be adjusted to the participants’ *arm span* (wrist to wrist) in 10 mm steps.

A black curtain measuring 1650 × 650 mm was placed over the table, 800 mm from the front edge, to obscure the location of the ball during setup (see Fig. 1). Its lower edge was weighted by a horizontal metal bar and extended by a 100 mm border of fringes, touching the top of each lane. Retro-reflective markers were placed on the table and on top of the curtain (see Fig. 1) to ensure perfect alignment of the setup and to calculate the moment the ball passed through the curtain.

A chair was centered near the front edge of the table (460 mm high, 240 mm leeway to the tabletop). Distance of the chair to the front edge could be adjusted by the participants. To the right and left of the chair, two open-fronted storage bins (300 × 190 × 145 mm) were placed (170 mm from the front edge, 600 mm apart), in which the ball had to be dropped after a catch (see Fig. 1).

Standardized golf balls (45.93 g weight, 42.67 mm diameter) were used as catching targets. The balls were covered with a retro-reflective film to track their position with an optical motion capture system.

### Preparation

Retro-reflective markers were attached to three bony landmarks on the left (L) and right (R) hands of the participants, respectively: *radial* (*L/R RS*) and *ulnar* (*L/R US*) *styloid process* and top of the third *metacarpal* (*L/R MC*). The *arm span* (between *LRS and RRS*) of the participants was measured in a t-pose (arms extended sideways and palms pointed forward).

The setup was individually adjusted to the arm span of each participant. To this end, distance between lanes was set to *1/8th of the measured arm span*. Calculated positions for each lane had to be rounded to full centimeters to match the mounting points on the table surface.

To adjust the distance to the table, participants were seated on the chair in an upright position and asked to place the hands on top of the table, and the knuckles at a height with the front edge. Participants then had to move forward/backward until the upper arms were aligned vertically.

### Procedure

The experiment was split into two tasks. A task consisted of either ten (Task 1) or twenty (Task 2) sequences of nine trials. A trial was defined as the unimanual catching of one ball. Each trial started from an initial position, with the palms of the hands placed on the knees in a sitting, upright posture. A ball was placed on the end of a lane by an experimenter seated behind the curtain. As soon as the ball passed the curtain, participants had to (1) choose the hand that felt most natural for catching the ball, (2) catch the ball with the chosen hand before it reached the end of the lane, (3) drop the ball into the respective bin (e.g., the left bin if the ball was caught with the left hand), and (4) return to the initial position.

Before the experiment started, participants executed three trials (random lanes) for training.

In Task 1, the participant performed ten randomized sequences of the nine lanes (9 lanes × 10 repetitions: 90 trials). For the randomized sequences, a pseudo-random list (Mersenne twister algorithm; Matsumoto and Nishimura 1998) was created before the experiment. From this list, the experimenter selected the next lane as soon as the ball was dropped into the bin.

In Task 2, the participants performed twenty ordered sequences of the nine lanes, ten leftward and ten rightward sequences, respectively (2 directions × 9 lanes × 10 repetitions: 180 trials). The sequence of directions was randomized. When placing the first ball in the lane, the experimenter announced the direction (‘from left to right’/‘from right to left’).

The order of both tasks was counterbalanced across participants. Participants had a resting period of 30 s between sequences (while the experimenter retrieved the balls) and of 5 min between tasks (and after the first 10 sequences of Task 2). The entire experiment lasted approximately 45 min.

### Data analysis

Movement data were recorded by a Vicon MX (Vicon Motion Systems, Oxford, UK) motion capture system. Marker trajectories were reconstructed in Vicon Nexus 2.6.1, labeled manually, and exported to MATLAB (2015a, The MathWorks, Natick, MA) for data analysis. Occasional gaps in the ball trajectories (when passing below the curtain) were filled with a custom-made algorithm that took the constant acceleration and linear trajectory enforced by the lane into account. Movement data were used only to automatically identify the lane, the selected hand for catching, and to measure reaction time (RT) in each trial.

For each trial, the moment the front edge of the ball passed below the curtain was measured based on the curtain marker and ball trajectories. To this end, we determined the first frame in which the center of the ball passed a virtual threshold 21 mm (half the ball’s diameter) in front of the curtain.

To determine the selected hand for each trial, the average absolute velocity of the *capitulum* markers (*L/R MC*) from (1) the moment the ball passed the curtain until (2) the moment the ball disappeared (in the hand) was calculated. The hand with the higher average velocity was automatically labeled as the catching hand.

RT was calculated as the time difference between (1) the moment the ball passed the curtain and (2) movement initiation, defined as the first frame in which the catching hand’s absolute velocity exceeded 5% of its maximum absolute velocity during the reaching movement.

For each participant, ten trials per condition (randomized, leftward, rightward) and lane were used to calculate the probability of a right-handed catch. The average probability of a right-handed catch (%RH) and of a left-handed catch (%LH) in each condition and group was used for further analyses.

To test for hysteresis, paired, two-sided Wilcoxon signed rank tests were calculated on the %RHs in the left- and rightward sequences. To test for a shift of the POC within each participant group, paired, two-sided Wilcoxon signed rank tests on the %RH vs. %LH in the randomized and ordered conditions were calculated. The Holm–Bonferroni (HB) correction was applied to the *p* values. To compare the magnitude of the POC shift, an unpaired, two-sided Wilcoxon rank sum test on the %RH in the right-handed vs. the %LH in the left-handed group was calculated in the randomized and ordered conditions.

To test for differences in RT as a function of planning condition, the RTs from the ordered conditions (left-/rightward) were separated by hand and planning condition: (1) trials with no re-planning (reuse of the same hand), (2) trials with some re-planning (directly before a hand switch), and (3) trials with full planning from scratch (at the beginning of a sequence). A repeated measures analysis of variance was calculated on the separated RTs, with planning ‘condition’ and ‘hand’ as within-subject and ‘handedness’ as a between-subject factor.

To address the higher inconsistency of hand selection in left-handers (Gonzalez and Goodale 2009), we conducted all analyses twice, once with 27 and once with 19 participants in the left-handed group (excluding the 8 ambidextrous participants). Results did not differ qualitatively for all but one test. We, therefore, only report the results for the full left-handed group and the single difference found in the reduced group. The full results section for the reduced group is available as an electronic supplement.

## Results

To test if participants persisted on using the previously selected hand [resulting in different points-of-change (POCs) in the left-/rightward sequences], a paired, two-sided Wilcoxon signed rank test was calculated on the average probabilities of a right-handed catch in the left- and rightward sequences.

**Fig. 2:**
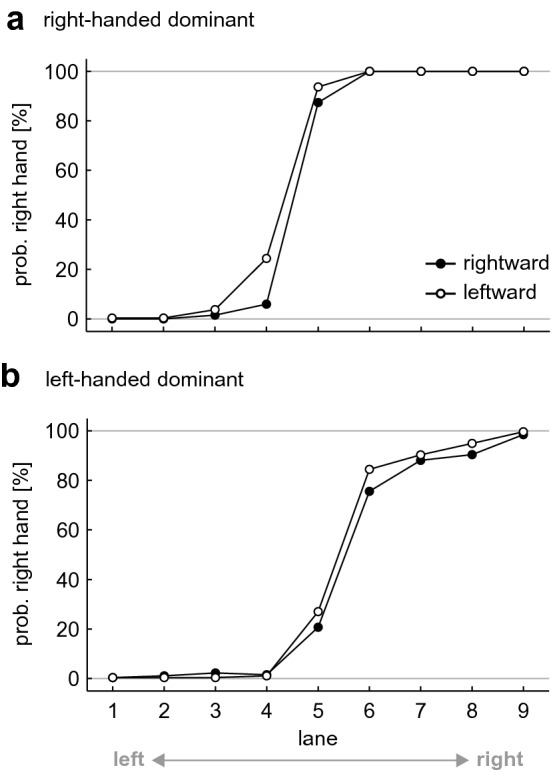
Probability of a right-handed catch in the ordered sequences for **a** right- and **b** left-hand-dominant participants, split by 'lane'. Each data point represents the average across the factors 'repetition' and 'participant'. Data were separated by 'direction' (black circles for rightward, white circles for leftward sequences)

For right-handed participants, results showed a significant difference in probabilities, *Z* = 2.682, *p* = 0.007, *r* = 0.365. Participants chose the right hand significantly more often in the leftward sequences (58.1 ± 6.3%) than in the rightward sequences (55.0 ± 4.4%), indicating that they persisted on the previously selected hand (see Fig. 2a).

For the full left-handed group (27 participants), results showed no significant difference, *Z* = 1.799, *p* = 0.072, *r* = 0.245. The probability of a right-handed catch was similar in the left- (44.3 ± 9.4%) and rightward (42.1 ± 11.2%) sequences (see Fig. 2b).

For the reduced left-handed group (19 participants), in contrast, we found a significant difference in probabilities, *Z* = 2.797, *p* = 0.005, *r* = 0.454. The truly left-handed participants chose the right hand significantly more often in the leftward sequences (42.3 ± 10.3%) than in the rightward sequences (38.8 ± 11.6%), indicating that they persisted on the previously selected hand (see electronic supplement, Fig. S1b).

To test for a significant shift of the POC as a function of handedness, we calculated a paired, two-sided Wilcoxon signed rank test on the average probabilities of a right-handed vs. a left-handed catch in the randomized and ordered condition in both groups. For the ordered condition, probabilities of the left- and rightward sequences were averaged for each lane. The Holm–Bonferroni (HB) correction was applied to the *p* values to adjust for family-wise errors due to multiple testing.

**Fig. 3:**
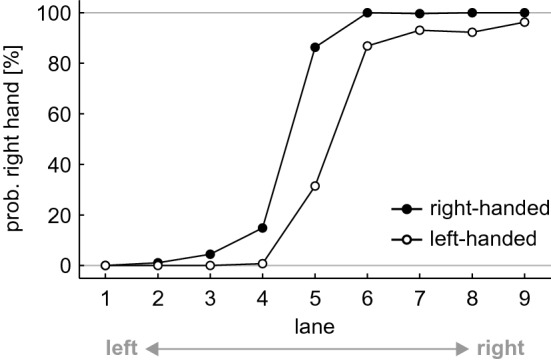
Probability of a right-handed catch in the randomized sequences, split by 'lane'. Each data point represents the average across the factors 'repetition' and 'participant'. Data were separated by 'group' (black circles for right-hand-dominant, white circles for left-hand-dominant participants)

For the right-handed participant group, results showed a significant difference in probabilities in the randomized, *Z* = 4.517, *p*_HB_ < 0.001, *r* = 0.615, and in the ordered, *Z* = 4.406, *p*_HB_ < 0.001, *r* = 0.600, condition. Participants chose the right hand (56.3 ± 4.7%, 56.5 ± 4.8%) significantly more often than the left hand (43.7 ± 4.7%, 43.5 ± 4.8%), indicating a clear shift of the POC towards the left (cf. Figs. 2a, 3).

For the left-handed participants, we also found a significant difference in probabilities in the randomized, *Z* = 3.495, *p*_HB_ < 0.001, *r* = 0.476, and in the ordered, *Z* = 3.805, *p*_HB_ < 0.001, *r* = 0.518, condition. Participants chose the right hand (44.5 ± 9.5%, 43.2 ± 9.8%) significantly less often than the left hand (55.5 ± 9.5%, 56.8 ± 9.8%), indicating a clear shift of the POC to the right (cf. Figs. 2b, 3).

The POC was shifted to the non-dominant side in both participant groups.

To test whether the shift of the POC towards the non-dominant side was similar in magnitude in left- and right-handed participants, we calculated an unpaired, two-sided Wilcoxon rank sum test on the probability of a right-handed catch in the right-handed vs. the probability of a left-handed catch in the left-handed group.

Right-handed participants chose the right hand as often (56.3 ± 4.7%, 56.5 ± 4.8%) as left-handed participants chose the left hand (55.5 ± 9.5%, 56.8 ± 9.8%), both in the randomized, *Z* = 1.990, *p*_HB_ = 0.093, *r* = 0.271, and ordered condition, *Z* = 1.356, *p*_HB_ = 0.175, *r* = 0.185. The magnitude of the POC shift was similar in both groups (cf. Figs. 2, 3).

To compare RTs of different planning conditions (no re-planning, some re-planning, full planning), a repeated measures analysis of variance was calculated on the aggregated RTs from each planning condition in the ordered (left-/rightward) sequences. Planning ‘condition’ and ‘hand’ (right/left) were within-subject factors, ‘handedness’ was a between-subjects factor. Neither the main effect nor any of the interactions including ‘handedness’ were significant. RTs did not differ between groups.

**Fig. 4:**
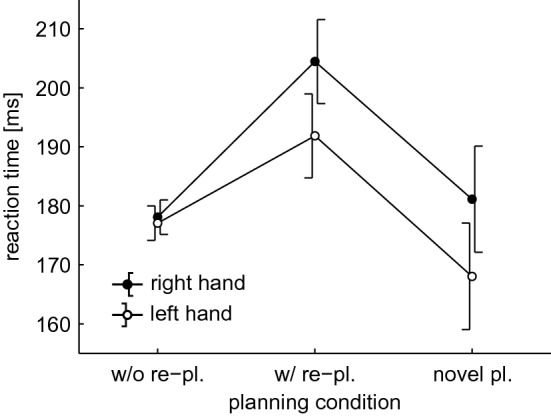
Reaction time for trials without re-planning, with some re-planning, and with novel planning. Each data point represents the average across the factor 'participant' and across all trials matching the planning condition. Data were separated by 'hand' (black circles for right hand, white circles for left hand). Error bars indicate 95% confidence intervals, adjusted for the within-subject variance of factor 'hand'

There was a significant main effect of ‘hand’, *F*(1,52) = 4.168, *p* = 0.046, *η*_p_^2^ = 0.074. RTs in the right hand (187.9 ms) were longer than in the left hand (179.0 ms; see Fig. 4). We also found a significant main effect of planning ‘condition’, *F*(2,104) = 10.221, *p* < 0.001, *η*_p_^2^ = 0.164. Contrary to our expectation, we did not find a significant linear contrast, *F*(1,52) = 0.234, *p* = 0.630, *η*_p_^2^ = 0.004, but a significant quadratic contrast, F(1,52) = 24.767, *p* < 0.001, *η*_p_^2^ = 0.323. The quadratic contrast indicates that RT in trials with re-planning differed from the RTs in the other two conditions. Post hoc, paired *t *tests confirmed that RT in trials with re-planning (198.2 ms) was significantly longer than in trials without re-planning (177.6 ms), *t*(53) = 3.974, *p*_HB_ < 0.001, d = 0.541, and in trials with full novel planning (174.6 ms), *t*(53) = 4.205, *p*_HB_ < 0.001, *d* = 0.572 (see Fig. 4). The interaction of ‘hand’ and planning ‘condition’ was not significant.

## Discussion

In the current study, we asked whether a left-shift of the point-of-change (POC) observed for right-handed participants in a previous study (Rostoft et al. 2002) reflected (a) a left hemisphere advantage in the cognitive cost of motor planning, (b) a dominant hemisphere advantage in the mechanical cost of motor execution, or (c) a combination of both factors. To distinguish between these alternatives, we replicated the study by Rostoft et al. with a left- and a right-hand-dominant, adult participant group. Participants had to catch a ball that was rolled towards them in one of nine lanes mounted on an inclined tabletop, choosing the hand that felt most natural. Lanes were varied in randomized and ordered (left-/rightward) sequences.

A left-shift of the POC across the mid-line, as observed in the study by Rostoft et al. (2002), implies that contralateral reaching movements were executed with the right hand. Contralateral reaches have several disadvantages, including longer reaction times (RTs), longer movement durations, and lower precision (Carey et al. 1996; Carson et al. 1992; Hoptman and Davidson 1994). For these movements to be viable, some reduction in total movement cost needs to compensate for the disadvantages. Two potential candidates were put to the test in the current study: the cognitive cost of motor planning and the mechanical cost of motor execution.

According to Janssen et al. (2009, 2011), the left hemisphere is specialized in motor planning. Such specialization increases the efficiency of the underlying neural circuits (Haslinger et al. 2004; Hund-Georgiadis and von Cramon 1999; Jäncke et al. 2000; Krings et al. 2000; Meister et al. 2005) and, thus, reduces the cognitive cost of motor planning in the right hand. According to Sainburg (2002), the dominant hemisphere is superior in the control of inertial dynamics: the dominant limb executes reaching movements with a fraction of the total torque impulse required for the same movement by the non-dominant limb (Coelho et al. 2013; Sainburg 2002). In right-handed participants, this should reduce the mechanical cost of motor execution in the (dominant) right hand.

In right-hand-dominant participants, both factors act agonistically to reduce the total motor cost of right-handed reaches and, thus, to compensate for the disadvantages of contra lateral reaching. In left-handed participants, in contrast, the mechanical cost of movement execution should be lower in the (dominant) left hand and, thus, act antagonistically to the higher cognitive cost. By testing both left- and right-handed participants, we could isolate the effect both cost factors. If the cognitive cost alone was relevant for the POC shift, we expected a left-shift in both the right- and the left-handed group. If only the mechanical cost was relevant, we expected a left-shift in the right-handed but a right-shift in the left-handed group.

Results showed a significant left-shift of the POC in the right-handed and a significant right-shift in the left-handed group, both in the ordered and randomized condition. This implies that contralateral reaches are viable primarily due to their lower mechanical cost of motor execution. To test whether cognitive cost was relevant to the POC shift as well, we compared the magnitude of the POC shift in both groups. As cognitive and mechanical costs act agonistically (sum of effects) in right-handed and antagonistically (difference of effects) in left-handed participants, a difference in magnitude reflects twice the effect of the cognitive cost. Results showed no significant difference in the magnitude of the POC shift between groups, neither in the randomized nor in the ordered condition. This implies that cognitive cost does not affect hand selection at all.

Our results only support the idea of lower mechanical cost of motor execution in the dominant limb (Sainburg 2002). In a recent study on posture selection (Schütz and Schack 2019), we were unable to demonstrate this effect. Left- and right-handed participants executed a sequential drawer opening task with their dominant and non-dominant hand. We expected a smaller hysteresis effect in the dominant hand of both groups due to a lower mechanical cost of motor execution. Results showed a significant hysteresis effect, but no effects of hand or group. A main difference between the two studies was the motor task: Movements in the current study more closely resembled the ballistic reaching movements studied by Sainburg (2002). In the drawer task, the opening/closing phase of the drawer outweighed the ballistic approach phase, which might have shifted the focus from the perceived mechanical cost of the approach towards the perceived postural comfort in the opening/closing phase.

A left hemisphere specialization in motor planning (Janssen et al. 2009, 2011) was not found in the current study. It has mainly been supported by neuroimaging studies with right-handed participants (Haaland and Harrington 1996; Schluter et al. 2001, 1998), which results in a potential confound of a *left hemisphere* and a *dominant hemisphere* specialization. In the current study, a lower cognitive cost in the dominant hemisphere would act agonistically to the lower mechanical cost in the dominant hemisphere (independent of handedness) and, thus, would also result in the observed POC shifts. We therefore cannot exclude this possibility. Kim et al. (1993), however, tested left- and right-handed participants and found that the left hemisphere was active during both contra and ipsi lateral finger movements (irrespective of handedness). This finding supports a left hemisphere specialization in motor planning.

In behavioral studies, on the other hand, only the study by Janssen et al. (2009, 2011) showed a left hemisphere advantage in motor planning. Other studies were unable to reproduce Janssen’s results: In unimanual (Hughes and Franz 2008; Weigelt et al. 2006) and bimanual (Hughes et al. 2011) reach-and-place tasks similar to the task of Janssen et al. (2009, 2011), no difference in anticipatory posture planning was found between the left and right hand. However, the task by Janssen et al. (2009, 2011) had considerable precision requirements, which were absent in the other behavioral studies and in our experiment. Stone et al. (2013) found that right-hand use in a block-stacking task increased with the precision requirements. If the prevalence of the left-hemisphere specialization is indeed affected by task complexity, the catching task used in the current study might simply not have required enough higher level motor planning.

Even though hemispheric differences in cognitive cost could not be observed, hand selection was still affected by the cognitive cost of motor planning: results showed a significant hysteresis effect in the right-handed group, i.e., a persistence on the previously selected hand. This result is in accordance with previous studies on limb selection (Rostoft et al. 2002; Weiss and Wark 2009) and supports the idea that motor planning is associated with a cognitive cost, which is reduced by a reuse of the former plan (Rosenbaum and Jorgensen 1992). The size of the hysteresis effect (3.1%) seems smaller than in the study by Rostoft et al. (2002), even though no directly comparable values were reported. This might indicate that time pressure was less for our adult participants, even though the steepness of the incline had been doubled.

When testing the full left-handed group (self-report, 27 participants), the hysteresis effect did not reach significance. If the 8 ambidextrous participants (Edinburgh inventory) were removed from the group, results showed a significant hysteresis effect similar in size (3.5%) to that of the right-handed group. This finding is in line with previous reports that found a larger inconsistency in hand selection for left-handers (Gonzalez and Goodale 2009; Papadatou-Pastou et al. 2013). Presumably, the larger variance in hand selection caused by the 8 ambidextrous participants prevented the hysteresis effect from reaching significance.

The reaction time (RT) analysis demonstrated shorter RTs for the left-handed reaches, independent of handedness. This result was expected, as it is in accordance with previous studies (Boulinguez et al. 2000, 2001; Carson et al. 1995, 1992). The basis of this RT advantage is still being debated: it has either been attributed to a faster spatial parameterization of movements in the right hemisphere (Carson et al. 1995; Mieschke et al. 2001) or to the right hemisphere’s special role in visual attention (Barthelemy and Boulinguez 2001; Corbetta et al. 2000; Posner et al. 1987). Our current results cannot be used to support one of these competing hypotheses over the other.

We also expected a linear increase of RT for increasing planning demands: (1) if no re-planning was required, (2) if some re-planning was required, and (3) if full planning was required. In the literature, higher planning costs are associated with longer RTs (Diedrichsen et al. 2001, 2003; Spijkers et al. 1997). Instead, results showed a significant quadratic contrast, with the highest RTs directly before a hand switch (some re-planning required). This result might reflect hand selection: Carson et al. (1995) had participants execute pointing movements with their left or right hand. Hand was cued either before or in parallel to the target. Results showed longer RTs (~ 140 ms) if hand and target were selected in parallel. Thus, the longer RTs we found directly before a hand switch (~ 20 ms) might reflect the uncertainty in hand selection, which is high in the proximity of the POC and lower at the start of a sequence.

In conclusion, the current study extends previous research on hand selection in a sequential reaching task. It shows that shifts of the POC across the midline that were observed in previous studies can be attributed to a lower mechanical cost of motor execution in the dominant hand, which compensates for the disadvantages of the contralateral reaching. This finding supports the *dynamic-dominance hypothesis* by Sainburg (2002). In contrast, POC shifts seem unaffected by hemispheric differences in the cognitive cost of motor planning, as suggested by Janssen et al. (2009, 2011).
